# Screening and Characterization of *Lactiplantibacillus plantarum* WYP with Histamine-Degrading Activity: A Probiotic Candidate Assessed Based on Phenotyping Experiments and Whole-Genome Sequencing

**DOI:** 10.3390/foods15101763

**Published:** 2026-05-16

**Authors:** Yaping Wang, Haiqian Xu, Yanyan Huang, Langhong Wang, Mansheng Wang, Qinglin Sheng

**Affiliations:** 1 College of Food Science and Technology, Research Center of Food Safety Risk Assessment and Control, Northwest University, Xi’an 710069, China; wyping1023@163.com; 2 Guangdong Provincial Key Laboratory of Intelligent Food Manufacturing, School of Food Science and Engineering, Foshan University, Foshan 528225, China; 15819320870@163.com (H.X.); huang_yanyan@fosu.edu.cn (Y.H.); wlhong@fosu.edu.cn (L.W.); 3 Institute of Bast Fiber Crops, Chinese Academy of Agricultural Sciences, Changsha 410205, China

**Keywords:** *Lactiplantibacillus plantarum*, whole-genome sequencing, histamine degradation, multicopper oxidase, food safety, probiotic potential

## Abstract

This study isolated and characterized *Lactiplantibacillus plantarum* WYP from naturally fermented pineapple peel residues. The strain exhibited a potent in vitro histamine degradation rate of 78.63% and demonstrated multiple probiotic properties, including acid and bile salt tolerance, simulated gastrointestinal fluid resistance, antimicrobial activity against foodborne pathogens, and in vitro cholesterol-lowering ability. Whole-genome sequencing revealed a 3.34 Mb circular genome encoding 3200 genes. Genomic analysis elucidated a multidimensional “Prevention–Promotion–Utilization” (PPU) strategy for histamine regulation: prevention via the absence of histidine decarboxylase (*hdc*) genes; promotion of degradation via multicopper oxidase (e.g., *cueO*) and amine oxidase systems; and utilization through downstream aldehyde metabolism and redox homeostasis genes. Safety assessments confirmed the strain’s non-hemolytic nature, absence of harmful metabolite production, and no detectable risk of acquired antibiotic resistance gene transfer. The integration of phenotypic and genomic evidence positions LPWYP as a promising probiotic candidate for mitigating biogenic amines in fermented foods.

## 1. Introduction

Biogenic amines (BAs) are nitrogenous organic compounds commonly generated in fermented foods via the microbial decarboxylation of amino acids [1]. At low concentrations, BAs are generally regarded as safe and contribute to essential physiological functions, including the modulation of neurotransmission and vascular tone. However, when dietary intake exceeds a certain threshold, these compounds can induce a spectrum of toxicological effects, such as headaches, nausea, and anaphylactic shock [2,3]. Common BAs found in fermented products include tryptamine, phenethylamine, putrescine, cadaverine, histamine, tyramine, spermidine, and spermine [4]. Among these, histamine poses the greatest acute food safety risk [5]. Its diverse toxicological effects—stemming from its dual endogenous roles as a neurotransmitter and hormone—range from dermatological and gastrointestinal disturbances (e.g., erythema, pruritus, diarrhea, vomiting, and abdominal pain) to cardiovascular reactions (e.g., hypotension, dizziness, and tachycardia) [6,7,8].

The accumulation of BAs in fermented foods is primarily attributed to the metabolic activities of microorganisms harboring amino acid decarboxylases. Given the complexity of microbial metabolism and fermented matrices, conventional physicochemical interventions such as thermal processing and chemical preservatives have shown limited efficacy in mitigating BA formation. Moreover, the use of chemical methods also conflicts with “clean” and “green” labeling claims [9]. These limitations have prompted growing interest in biological strategies that can actively degrade BAs during fermentation, thereby enhancing both food safety and product quality.

Lactic acid bacteria (LAB) have emerged as promising candidates for BA degradation, owing to their generally recognized as safe (GRAS) status and their capacity to produce amine-oxidizing enzymes, such as multicopper oxidases (MCOs) and amine oxidases, which facilitate the breakdown of BAs into less harmful metabolites. Over the past two decades, numerous LAB strains with BA-degrading capacity have been isolated from conventional fermented products, including fermented meats, dairy products, and plant-based fermented foods [10,11,12]. However, most of these studies have relied primarily on phenotypic assays, providing limited insight into the genetic underpinnings of BAs degradation. To address these gaps, this study employed a comprehensive phenotype–genotype approach to isolate and identify a novel Lactobacillus strain capable of degrading histamine. Unlike many previous studies that relied primarily on phenotypic screening, our strategy combines in vitro functional assays (e.g., degradation rates, probiotic properties, and safety testing) with whole-genome sequencing (WGS) and comparative genomic analysis. This integrated framework aims not only to identify an efficient strain but also to elucidate the genetic blueprint underlying its bioamine-degrading activity.

Specifically, we focused on the under-explored field of natural fermentation of pineapple peel residues. Reports indicate that the crude protein content of pineapple peel is approximately 5.78% on a dry weight basis [13], which is significantly higher than that of other tropical fruit peels, such as papaya (3.50%) [14] and jackfruit (1%) [15]. This substrate is rich in protein and polyphenols and features a dynamic, high-stress environment. Theoretically, it provides abundant nutritional conditions for lactic acid bacteria with robust and versatile metabolic networks, making it a promising source for identifying strains with enhanced amine-metabolizing capabilities [16]. The primary objectives of this study are: (1) to isolate and screen lactic acid bacteria capable of degrading histamine from this substrate; (2) comprehensively evaluate the probiotic properties and safety of the selected strains through phenotypic experiments; (3) elucidate their phylogenetic position, genomic characteristics, and potential genetic determinants for histamine degradation identified through computational simulation analysis via whole-genome sequencing; (4) propose the genetic mechanisms underlying their ability to alleviate histamine poisoning. We anticipate that the combined phenotypic and genomic evidence will provide a solid foundation for positioning this strain as a promising probiotic candidate and offer new insights into the molecular basis of amine degradation in fruit-derived lactic acid bacteria.

## 2. Materials and Methods

### 2.1. Materials and Strains

*Lactiplantibacillus plantarum* DMDL 9010 (LP9010, CGMCC 5172) was used as the positive control strain, and *Escherichia coli* ATCC 35150 (EC35150, GDMCC 1.707), *Staphylococcus aureus* ATCC 25923 (SA25923, GDMCC 1.174), *Listeria monocytogenes* ATCC 19115 (LM19115, GDMCC 1.347), and *Salmonella enteritidis* ATCC 9120 (SE9120, GDMCC 1.1114) were used as the indicator strains. All bacterial strains were purchased as lyophilized cultures from the Microbial Culture Collection Center of Guangdong Institute of Microbiology (Guangzhou, China). The LP9010 strain was cultured in MRS medium under facultative anaerobic conditions at 37 °C, while the indicator strains were activated in LB broth at 37 °C for 18 h with shaking at 180 r/min.

Histamine dihydrochloride was purchased from Shanghai Aladdin Company Limited (Shanghai, China). All other reagents were commercially available analytical grade or biochemical grade. Organic solvents, water, and standards used in high-performance liquid chromatography (HPLC) analysis were chromatographic grade, while other reagents were analytical grade or biochemical grade.

### 2.2. Screening of LAB with Biogenic Amine Degradation Effect

#### 2.2.1. Isolation, Purification and Preliminary Identification of LAB

Following the method described by Li [17], samples of fermented pineapple peel residue were serially diluted in physiological saline (10^−1^–10^−8^) and spread onto calcium carbonate agar plates, which were then incubated anaerobically at 37 °C for 48 h. Colonies exhibiting distinct calcium-dissolving zones were selected and streaked onto MRS agar plates for purification. Isolates that were hydrogen peroxidase-negative and Gram-positive were preliminarily identified as Lactobacillus and stored at −80 °C in tubes containing 25% glycerol.

#### 2.2.2. Screening of LAB with Non-Biogenic Amine Production and Histamine Degraation Capability

Following the method described by Sun [18], the activated strains were streaked onto a double-layer chromogenic medium. After 3 days of incubation, a chromogenic reagent was added and the plates were observed for 5 min; a purple color indicated a positive result for biogenic amines, while a yellow color (or no color change) indicated a negative result. Negative strains were selected.

Histamine degradation capacity was determined in accordance with Chinese National Standard GB 5009.208-2016 [19]. Inoculate the strain (1 × 10^9^ CFU/mL) into MRS medium containing 500 mg/L histamine, incubate at 37 °C for 48 h, centrifuge to collect the supernatant, derivatize the sample, and determine the histamine content using HPLC (Agilent 1260, California, USA) to calculate the degradation rate. Strains with strong degradation capabilities were selected and stored at −80 °C in 25% glycerol. The degradation rate is calculated using the following formula:

(1) $$\mathrm{Degradation}\;\mathrm{rate}\;\mathrm{of}\;\mathrm{histamine}\;\left(\%\right)\;=\;(T_{1}-T_{2})/T_{1}\;\times \;100\;$$

where T_1_ denotes the histamine concentration in the initial solution (mg/L), and T_2_ denotes the residual histamine concentration after the degradation process (mg/L).

#### 2.2.3. Acid and Bile Salt Tolerance Test

The activated strains were inoculated separately into MRS medium adjusted to different pH levels (2.0, 4.0, 6.0) using HCl or NaOH, as well as into MRS medium containing different concentrations of bile salts (0.1%, 0.3%, 0.5%, 1.0%). The inoculation volume was 2% (*v*/*v*), and the plates were incubated anaerobically at 37 °C for 24 h. Live bacteria were counted on MRS agar plates using the dilution plate count method, and the survival rate was calculated.

### 2.3. Strain Identification and Characterization

#### 2.3.1. Identification of LAB by 16S rRNA

The 16S rRNA sequencing of candidate strain was carried out by Guangzhou Aiji Biotechnology Co., Ltd. (Guangzhou, China). The phylogenetic tree was constructed with Mega 11 software.

#### 2.3.2. Morphological Observation

The morphological characteristics of the LAB were observed and screened using standard microbiological methods. The activated bacterial solution was inoculated into MRS broth, and after overnight incubation at 37 °C, streaking was performed onto MRS agar plates to observe the colony morphology. Meanwhile, according to the method described by Zhang [20], the microscopic morphology of the strains was observed using a scanning electron microscope (Quattro S, Thermo Fisher Scientific, Waltham, MA, USA). Additionally, the activated bacterial suspension was stained with Gram’s stain, and the staining results were observed under an optical microscope (Mshot MF53, Guangzhou Micro-Pic Technology Co., Ltd., Guangzhou, China).

#### 2.3.3. Physiological and Biochemical Assays

Conventional microbiological methods were used to determine the physiological and biochemical characteristics of LAB, including glucose-phosphate peptone broth (acetomethyl methanol and methyl red tests), hydrogen sulfide production, carbohydrate fermentation, gelatin liquefaction, and starch hydrolysis. The carbon source utilization profile was determined using the API 50 CHL kit (BioMérieux, Lyon, France): fresh bacterial suspensions were inoculated onto test strips containing 49 types of carbohydrates, incubated at 37 °C for 24 and 48 h, and fermentation results were read using the API system.

#### 2.3.4. Growth Curve and Acid Production Curve Measurements of LAB

Inoculate MRS broth with a 2% (*v*/*v*) suspension of activated bacterial culture, incubate at 37 °C, and take samples every 2 h to measure OD_600_ and pH until the culture reaches the stationary phase.

### 2.4. Whole-Genome Sequencing and Functional Analysis

#### 2.4.1. Genomic DNA Extraction, Library Construction and Sequencing

This study used LPWYP as the test material. Approximately 200 mg of bacterial cells were taken, frozen in liquid nitrogen, and ground into a powder. Since this bacterium is Gram-positive, it was first treated with lysozyme at 37 °C for 30 min, followed by the addition of CTAB lysis buffer and vortexing to mix thoroughly. The mixture was incubated at 65 °C at 400–1400 rpm for 60 min, cooled to room temperature, and then centrifuged at 12,000 *g* for 5 min. The supernatant was aspirated, and an equal volume of phenol/chloroform/isoamyl alcohol (25:24:1) was added. The mixture was shaken to mix, then centrifuged at 12,000 *g* for 10 min. Transfer the supernatant to a new tube, add 2/3 volume of isopropanol and 100 μL of sodium acetate, gently invert to mix, and let precipitate at −20 °C for at least 2 h. Centrifuge at 18,213 *g* for 15 min at room temperature, discard the supernatant, wash the pellet with 1 mL of 75% ethanol, and centrifuge again for 3 min. Discard the ethanol, centrifuge briefly, and aspirate the residual liquid. Allow it to air-dry at room temperature for 3–5 min, then add an appropriate volume of TE buffer to dissolve the genomic DNA.

Use the MGIEasy Universal DNA Library Preparation Kit (MGI-Shenzhen, Shenzhen, China) to construct a library from the genomic DNA of LPWYP. After DNA fragmentation and screening, perform end repair, 3′ A-tailing, and adapter ligation, followed by PCR amplification. After the library passes quality control, denature, circularize, and perform rolling circle replication to form DNA nanoballs, then perform PE150 sequencing on the DNBSEQ-G400 sequencing platform (MGI-Tech., Shenzhen, China).

#### 2.4.2. Genome Assembly and Annotation

Raw sequencing data were processed using SOAPnuke 1.5.6 for quality control to obtain clean reads. The specific steps included: removing adapter sequences, reads with ≥40% of bases having a quality score ≤ 20, reads with ≥10% N bases, and duplicate sequences. SPAdes (v3.9.0) was used to perform de novo assembly of the Clean Reads, and the optimal assembly result for the LPWYP genome was obtained through local optimization.

Genome annotation followed the following workflow: Coding genes were predicted using Glimmer 3.02 [21]. Non-coding RNAs were predicted using RNAmmer 1.2 (rRNA) [22], tRNAscan-SE 1.3.1 (tRNA) [23], and Infernal (aligned against the Rfam 9.1 database) (sRNA) [24]. Repetitive sequences were identified using Tandem Repeats Finder 4.04 [25]. To elucidate their functional potential, the amino acid sequences of the predicted genes were aligned using Diamond against KEGG (101), COG (25 November 2020), Swiss-Prot (release-2021_04), CARD (v3.0.9), CAZy (13 October 2021), VFDB (25 November 2021), and the T3SS effector protein (1.0) database. The whole-genome sequence data for LPWYP were submitted to GenBank under access number PRJNA1459682.

### 2.5. Comparative Genomic Analysis

Comparative genomic analysis was performed between the sequenced LAB strain and several reference *Lactiplantibacillus plantarum* strains, including *L. plantarum* WCFS1 (NCBI accession: NC_004567), *L. plantarum* ZJ316 (NCBI accession: CP004082), *L. plantarum* JDM1 (NCBI accession: NC_012984), *L. plantarum* 16 (NCBI accession: NC_021514), and *L. plantarum* NCU116 (NCBI accession number: NZ_CP016071). Whole-genome alignments were conducted using MUMmer 3+ software (http://mummer.sourceforge.net/, accessed on 3 December, 2025) to identify structural variations and genomic rearrangements. The resulting comparative data were analyzed to elucidate the phylogenetic relationships and evolutionary dynamics among the strains, providing insights into the genetic basis of strain-specific adaptations.

### 2.6. Gene Annotation Related to Histamine Degradation

Genes potentially involved in the histamine-lowering capability of the screened strain were identified through sequence alignment against the COG, KEGG, and Pfam databases. The biogenic amine metabolic pathways in the screened strain were then mapped by integrating KEGG pathway maps with the obtained gene annotation information.

### 2.7. Safety Analysis of the Strain and Annotation of Related Genes

#### 2.7.1. Detection of Potentially Harmful Metabolites and Annotation of Related Genes

The screened LAB strain was screened for potentially harmful metabolites and evaluated using methods including indole production, nitroreductase activity, amino acid decarboxylation, and hemolytic activity [26]. Genes associated with virulence were identified through VFDB analysis.

#### 2.7.2. Antibiotic Resistance Test

The antibiotic susceptibility of lactic acid bacteria strains was evaluated using the disk diffusion method according to the procedure described by Patel et al. with slight modifications. Briefly, bacterial cultures were adjusted to approximately 10^5^ CFU/mL (equivalent to 0.5 McFarland standard after appropriate dilution), and 100 μL of the suspension was uniformly spread onto MRS agar plates. Commercial antibiotic disks containing different antibiotics at standard concentrations were then placed on the agar surface using sterile forceps. The inoculated plates were incubated anaerobically at 37 °C for 24 h. After incubation, the diameters of inhibition zones were measured using a vernier caliper and expressed in millimeters (mm). Each assay was performed in triplicate.

### 2.8. Evaluation of Stress Tolerance and Annotation of Associated Genes

#### 2.8.1. Tolerance to Artificial Gastric and Intestinal Fluids

The tolerance of LAB strains to simulated gastrointestinal conditions was evaluated following the method described by Lu with minor modifications [27]. Artificial gastric fluid was prepared by supplementing sterile phosphate-buffered saline (PBS, pH 3.0) with pepsin at a final concentration of 0.3% (*w*/*v*). Artificial intestinal fluid was prepared by supplementing sterile PBS (pH 8.0) with trypsin at a final concentration of 0.1% (*w*/*v*). Both fluids were sterilized by filtration through a 0.22 μm membrane filter and preheated to 37 °C prior to use. Activated cultures were separately inoculated into artificial gastric fluid and artificial intestinal fluid to achieve a final concentration of approximately 10^9^ CFU/mL. The mixtures were incubated at 37 °C, and samples were collected at 0, 30, 60, 90, and 120 min for viable cell counting.

#### 2.8.2. Co-Aggregation Ability, Auto-Aggregation Ability, and Strain Surface Hydrophobicity Assays

The co-aggregation capacity of the LAB strain with pathogenic bacteria (EC35150, LM19115, SA9120, SE25923) was evaluated using the method described by Lu [27]. Equal volumes of LPWYP and each bacterial suspension were mixed, gently vortexed for 90 s, and incubated at 37 °C for 1 h and 4 h. Absorbance at 600 nm was measured, with strain LP9010 serving as the control.

The self-aggregation ability of the LAB strain was assessed using the method described by Li [28]. The brief procedure is as follows: Cell pellets were collected by centrifugation, washed with PBS, resuspended, and adjusted to an OD_600_ of approximately 0.6. The suspension was then incubated at 37 °C, and supernatants were collected at various time points to measure absorbance at 600 nm.

The cell surface hydrophobicity of the LAB strain was determined using the method described by Lu [29]. Genes associated with adhesion were annotated through a multi-database analysis (IPR/SWISS-PROT/COG/GO/KEGG/NR/T3SS).

#### 2.8.3. Antimicrobial Assay

The antimicrobial activity of the LAB strain against foodborne pathogens was evaluated using the agar well diffusion method (modified from the method described by Liu [30]). Pathogenic bacteria (EC35150, LM19115, SA 9120, SE25923) were adjusted to an OD_600_ of 0.5 and inoculated onto LB agar. Wells were prepared and filled with the tested LAB strain cell-free supernatant and bacterial suspensions. Diffusion was performed at 4 °C for 1 h, followed by incubation at 37 °C for 24 h. The diameter of the inhibition zone was measured using a Vernier caliper, with LP9010 serving as the control strain.

### 2.9. Statistical Analysis

All quantitative experiments in this study were independently repeated three times (biological replicates, *n* = 3), and data are presented as mean ± standard deviation. Statistical analysis was performed using Origin 2021 (OriginLab, Northampton, MA, USA) software. Specific methods were determined based on the experimental design: For single-factor between-group comparisons (e.g., survival rates under different pH conditions), one-way analysis of variance (ANOVA) was employed, preceded by Shapiro–Wilk and Levene tests to verify data normality and homogeneity of variances, respectively. If the parametric test conditions were met, Tukey’s post hoc test was used for multiple comparisons after significant differences were identified by ANOVA. For experiments involving two factors (time and treatment), such as simulating gastrointestinal fluid tolerance, a two-way ANOVA was employed. Data were analyzed for statistical significance using SPSS 26 (IBM, Armonk, NY, USA), with *p* < 0.05 indicating statistical significance.

## 3. Results and Discussion

### 3.1. Screening of Histamine-Degrading LAB

A total of 60 LAB strains were initially isolated from the residual debris of naturally fermented pineapple peels. To identify strains capable of degrading histamine without simultaneously producing biogenic amines, a primary screening was performed using a double-layer chromogenic medium. Strains that did not induce a color change (indicating a lack of biogenic amine production) were selected as negative candidates. This screening yielded 46 strains that were preliminarily classified as non-producers of biogenic amines.

**Figure 1:**
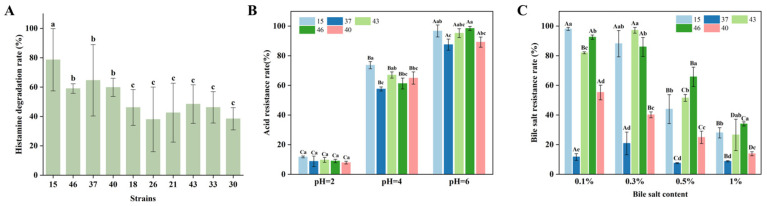
The histamine degradation capacities and tolerance of different strains. (**A**) represents the top 10 strains ranked by histamine degradation rate, (**B**) represents acid tolerance, and (**C**) represents bile salt tolerance rate. Distinct lowercase letters denote significant difference between experimental group and control group (*p* < 0.05). Distinct uppercase letters denote significant difference between different groups (*p* < 0.05).

The histamine degradation capacity of these 46 candidate strains was then quantitatively evaluated via HPLC. The top 10 strains ranked by histamine degradation rate are shown in Figure 1A and Table S1. Figure S1 presents the HPLC chromatograms of histamine degradation for the top 5 strains, and Figure S2 shows the histamine standard curve. It was observed that strain 15 exhibited the highest degradation efficiency, with a degradation rate of 78.63%. Based on this promising activity, the five strains with the highest degradation rates were subjected to further evaluation for their tolerance to acidic conditions and bile salts, the results of which are shown in Figure 1B,C, respectively. Strain 15 exhibited superior tolerance to both stresses compared to the other candidates. Consequently, given its outstanding histamine degradation capacity and robust stress tolerance, strain 15 was selected for comprehensive subsequent characterization.

### 3.2. Identification and Characterization of the Screened LAB

**Figure 2:**
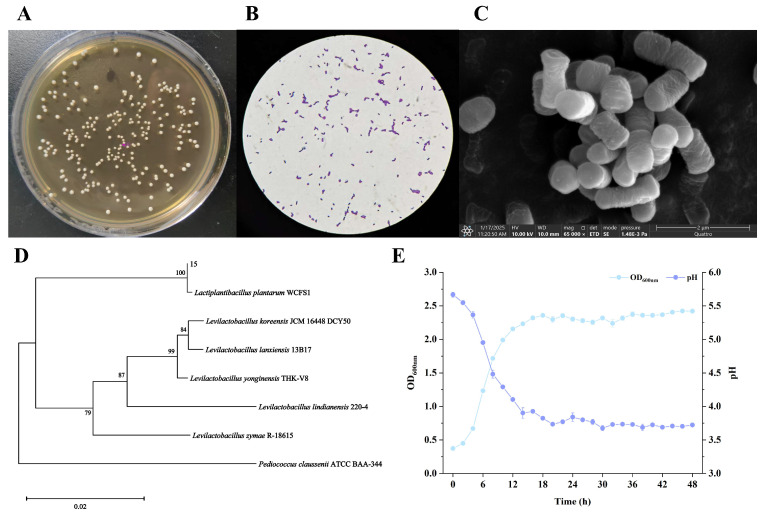
Identification and fermentation characterization of LPWYP. (**A**) is the colony morphology, (**B**,**C**) are the bacterial morphologies observed by OM and SEM, (**D**) is the phylogenetic tree, and (**E**) is the growth and acid production curve of LPWYP.

Strain 15 was phylogenetically identified by 16S rRNA gene sequencing. Analysis of the obtained sequence against the NCBI database revealed 100% identity with *Lactiplantibacillus plantarum* JCM1149. A phylogenetic tree was constructed to further clarify its taxonomic position (Figure 2D), which confirmed its clustering within the *L. plantarum* clade. Consequently, the strain was designated *Lactiplantibacillus plantarum* WYP (hereinafter referred to as LPWYP) and deposited in the Guangdong Microbial Culture Collection Center under accession number GDMCC 65359.

Colony morphology of LPWYP was observed on MRS agar. Colonies appeared circular, creamy white, smooth, moist, and approximately 1–2 mm in diameter (Figure 2A). Gram staining showed the cells to be rod-shaped and Gram-positive (Figure 2B). Scanning electron microscopy (SEM) further revealed that the cells existed as short rods, either singly or in short chains (Figure 2C). These morphological and staining characteristics are consistent with the typical description of *Lactiplantibacillus plantarum*.

Physiological and biochemical characterization of LPWYP was conducted using conventional biochemical tests. The strain tested negative for hydrogen sulfide production, starch hydrolysis, gelatin liquefaction, and the Voges–Proskauer (VP) reaction, but was positive in the methyl red (MR) test and in assays for fermenting various carbohydrates. These biochemical traits align with those characteristic of *L. plantarum* and further corroborate its species identification.

Growth kinetics and acidification profile of LPWYP were monitored over 24 h (Figure 2E). The strain exhibited a sigmoidal growth pattern: a brief lag phase (0–2 h) was followed by a rapid logarithmic growth phase (2–12 h), which then transitioned into the stationary phase after 12 h. Concurrently, the pH of the culture broth dropped sharply from approximately 6.5 to 3.94 by 12 h, and further to 3.7 by 24 h, demonstrating robust and sustained acid production [31]. A prolonged logarithmic phase is conducive to the accumulation of metabolites and adaptation to environmental stresses [32]. The acid-producing capacity of LPWYP is comparable to that reported for other *L. plantarum* strains, such as BHP03 [33]. In summary, the rapid growth and strong acidification capability of LPWYP underscore its potential utility as a starter culture for fermented foods.

### 3.3. Whole-Genome Sequencing and Analysis of LPWYP

**Figure 3:**
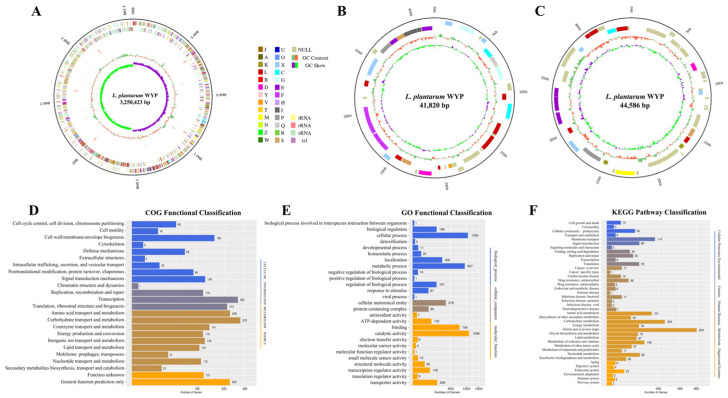
Genome profile of LPWYP and functional annotations in different databases. (**A**) is the chromosome map of LPWYP, (**B**) is the plasmid 1 ring map, (**C**) is the plasmid 2 ring map, (**D**) is the COG functional classification, (**E**) is the GO functional classification, and (**F**) is the KEGG functional classification.

The complete genomic structure and gene distribution characteristics of strain LPWYP are illustrated in Figure 3A–C and Table 1. The genome comprises a circular double-stranded chromosome and two plasmids, with a total size of 3,336,829 bp and a GC content of 45.57%. A total of 3200 coding sequences (CDSs) were identified, spanning 2,801,373 bp with an average length of approximately 875.43 bp per gene. The circular chromosome is 3,250,423 bp in length with a GC content of 44.61%, while plasmid 1 and plasmid 2 are 44,586 bp and 41,820 bp, with GC contents of 36.79% and 40.29%, respectively. Further genomic component prediction revealed the presence of 75 tRNA genes, 16 rRNA genes, and 4 sRNA genes. Additionally, 86 tandem repeats and 2 CRISPR sequences were identified, whereas no intact prophage regions were detected within the genome. Table S2 presents statistics on genomic coverage.

The predicted coding sequences (CDSs) of LPWYP were functionally annotated using the Clusters of Orthologous Groups (COG), Gene Ontology (GO), and Kyoto Encyclopedia of Genes and Genomes (KEGG) databases.

COG analysis assigned 2323 genes (72.59% of CDSs) to 24 functional categories (Figure 3D). Genes involved in metabolic processes constituted the most abundant category. Notably, 226 genes were associated with “amino acid transport and metabolism,” and 21 were linked to “secondary metabolite biosynthesis, transport, and catabolism,” indicating a genetic basis potentially relevant to biogenic amine metabolism. GO terms were assigned to 1897 genes (59.28%), categorizing them into biological processes, molecular functions, and cellular components (Figure 3E). Within biological processes, “cellular process” and “metabolic process” were predominant. For molecular function, “catalytic activity” and “binding” were the most highly represented terms, underscoring the strain’s enzymatic potential for substrate transformation. KEGG pathway mapping annotated 1846 genes (57.86%) across six major categories, with “Metabolism” being the largest (1549 genes) (Figure 3F). Key sub-pathways within metabolism included “carbohydrate metabolism” (252 genes) and “amino acid metabolism” (151 genes). Importantly, 28 genes were mapped to the “xenobiotics biodegradation and metabolism” pathway, which may encompass enzymes involved in biogenic amine degradation. Overall, these genomic analyses reveal that LPWYP possesses system-atic metabolic potential for degrading biogenic amines and other exogenous compounds.

### 3.4. Comparative Genomic Analysis of LPWYP

**Figure 4:**
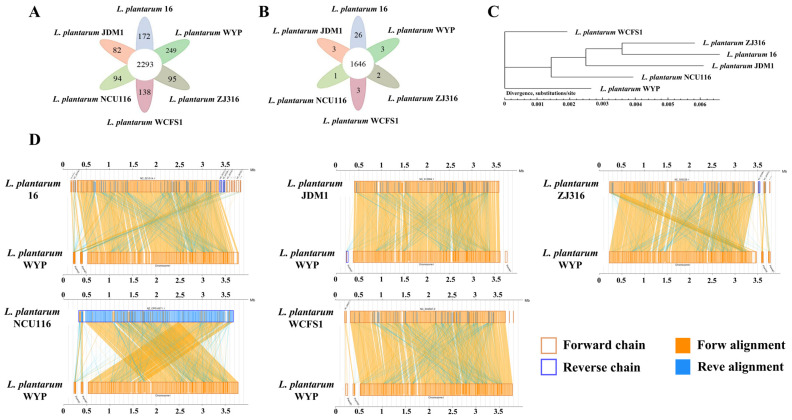
Comparative analysis of LPWYP and the other five *L. plantarum* strains. (**A**) represents the core genes and unique genes of the six strains; (**B**) is the number of homologous gene families; (**C**) is the phylogenetic tree based on the sequence similarity of 2293 core genes; (**D**) is the co-linearity analysis of LPWYP and the other five *L. plantarum* strains at the nucleic acid level.

Comparative genomic analysis was performed to elucidate the genetic relationship between LPWYP and five reference *L. plantarum* strains (16, JDM1, NCU116, WCFS1, and ZJ316). A phylogenetic tree constructed based on core-pan genome analysis further supported this finding, with LPWYP and LPWCFS1 clustering together, suggesting shared physiological characteristics and evolutionary history (Figure 4C).

Whole-genome synteny analysis was subsequently performed to assess genomic conservation at the macrostructural level. LPWYP exhibited high collinearity with WCFS1, 16, ZJ316, and JDM1, with conserved arrangement of major gene blocks, indicating relative stability of the core genome structure during evolution. Although local gene inversion was observed in comparison with NCU116, no large-scale insertions, deletions, or translocations were detected in the LPWYP genome, with only minor structural variations present (Figure 4D). In summary, these analyses confirm that LPWYP is a typical *L. plantarum* strain with a stable genetic background, demonstrating a high degree of genomic conservation and a particularly close phylogenetic relationship with strain WCFS1.

Although the LPWYP genome displays overall structural conservation, pangenome analysis against five reference *L. plantarum* strains uncovered significant uniqueness in its functional gene content. The analysis defined a core genome of 2293 genes shared among all six strains, representing approximately 70–75% of each strain’s gene repertoire and encoding essential cellular functions. Strikingly, LPWYP possessed 249 strain-specific genes—substantially more than any of the reference strains (Figure 4A)—suggesting the acquisition of unique genetic elements during its evolution, which may contribute to specialized metabolic capabilities or environmental adaptation.

Further examination of gene families identified 1936 families within the LPWYP genome. Of these, 1646 were core gene families common to all reference strains, forming the functional basis for conserved species-level activities. Notably, three gene families were found to be exclusive to LPWYP (Figure 4B). The presence of these unique families provides direct genomic evidence of functional diversification and the acquisition of distinct genetic traits. These exclusive elements may be linked to LPWYP’s adaptation to its native niche (fermented pineapple residue) and to specialized physiological functions, such as the metabolism of complex carbohydrates and the degradation of biogenic amines [34].

### 3.5. Genetic Analysis of LPWYP’s Histamine Degradation Potential

**Figure 5:**
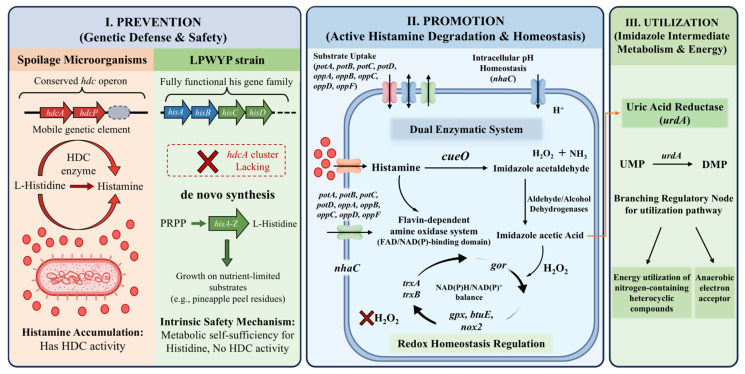
Histamine metabolic pathway in the LPWYP group. *cueO*: cuproxidase; HDC: histidine decarboxylase; *hdcA*: histidine decarboxylase, pyruvoyl type; *hdcP*: histidine/histamine antiporter; *hisA*: phosphoribosylformimino-5-aminoimidazole carboxamide ribotide isomerase; *hisB*: imidazolylglycerol-phosphate dehydratase; *hisC*: histidinol-phosphate aminotransferase; *hisD*: histidinol dehydrogenase; PRPP: phosphoribulose 1,5-bisphosphate; *trxA*, *trxB*: thioredoxin/thioredoxin reductase (NADPH); *gor*: glutathione reductase (NADPH); *gpx*/*btuE*: glutathione peroxidase; *nox2*: NADH oxidase (H_2_O-forming); *potA*: ATP-binding protein; *potB*, *potC*, *potD*: spermidine/putrescine transport system permease proteins; *oppA*: oligopeptide transport system substrate-binding protein; *oppB*, *oppC*, *oppD*, *oppF*: oligopeptide transport system permease proteins; *nhaC*: Na^+^:H^+^ antiporter, *NhaC* family; *urdA*: urocanate reductase; UMP: uridine monophosphate; DMP: dihydrouridine monophosphate; NAD(P): nicotinamide adenine dinucleotide phosphate; NADPH: nicotinamide adenine dinucleotide phosphate (reduced form).

The core finding of this study is the multidimensional metabolic strategy employed by LPWYP to regulate histamine levels; this study formally defines this framework as the “Prevention–Promotion–Utilization” (PPU) model (Figure 5). Unlike many LAB strains that possess only passive tolerance or partial degradation pathways, LPWYP exhibits a closed-loop system that combines safety with metabolic efficiency.

First, at the prevention level, LPWYP has established a robust genetic defense. The accumulation of histamine in the fermentation substrate primarily stems from the enzymatic conversion of L-histidine by microorganisms via histidine decarboxylase (HDC). This enzyme is typically encoded by the *hdcA* gene, which often belongs to a dedicated operon [35]. In many spoilage microorganisms, the *hdc* gene cluster is highly conserved and is often located on mobile genetic elements, enabling its widespread dissemination in contaminated food environments [36]. However, genomic analysis of the LPWYP strain reveals that it completely lacks the *hdcA* gene and its associated regulatory and transport genes, such as the *hdcP* gene encoding the histidine/histamine antiporter. Notably, LPWYP retains a fully functional his gene family (including *hisA*, *hisB*, *hisC*, *hisD*, *hisF*, *hisG*, *hisH*, *hisI*, and *hisZ*), which is responsible for the de novo synthesis of L-histidine starting from phosphoribulose 1,5-bisphosphate (PRPP) [37]. This genetic feature constitutes a robust intrinsic safety mechanism: despite lacking the enzymatic systems required to convert histidine into histamine, the strain maintains metabolic self-sufficiency for this essential amino acid, thereby supporting its growth on nutrient-limited substrates such as pineapple peel residues.

Secondly, in terms of degradation, LPWYP relies on a dual enzymatic system to actively reduce exogenous histamine concentrations. The primary driver of this activity is the multicopper oxidase (MCO) system, specifically the *cueO* homolog identified in the LPWYP genome. MCOs are a diverse family of enzymes that couple the four-electron reduction of molecular oxygen with water, while simultaneously performing single-electron oxidation of various substrates, including biogenic amines [38,39]. Studies on related strains such as L. plantarum LPZN19 have shown that the *cueO* enzyme promotes the oxidative deamination of histamine, yielding imidazoleacetaldehyde, ammonia (NH_3_), and hydrogen peroxide (H_2_O_2_) [38]. In addition to the multicopper oxidase, LPWYP may also establish a secondary degradation pathway via a flavin-dependent amine oxidase system. This enzyme contains a typical FAD/NAD(P)-binding domain and is capable of maintaining continuous histamine oxidation under varying redox conditions.

Notably, the LPWYP genome encodes multiple downstream aldehyde-metabolizing enzymes, including aldehyde dehydrogenases and alcohol dehydrogenases. These enzymes work synergistically to catalyze the further conversion of imidazoleacetaldehyde to imidazoleacetic acid, thereby completing the detoxification of histamine [40]. At the same time, reactive oxygen species (particularly H_2_O_2_) generated during histamine oxidation pose a potential oxidative stress to cells. LPWYP mitigates this side effect by establishing a comprehensive redox homeostasis regulatory system, including the thioredoxin system mediated by *trxA*/*trxB* [41], the glutathione reductase system encoded by gor [42], and antioxidant enzyme networks such as *gpx*/*btuE* and *nox2* [43,44]. This system efficiently scavenges peroxides and maintains intracellular NAD(P)H/NAD(P)^+^ balance, thereby ensuring the continuous progression of degradation reactions throughout different fermentation stages. Furthermore, LPWYP enhances histamine processing efficiency through various transport systems, such as the polyamine transport system encoded by *potABCD* and the oligopeptide transport system encoded by *oppABCDF*, which facilitate substrate uptake and the transport of metabolic intermediates [45,46]; simultaneously, ion transporters such as *nhaC* maintain intracellular pH homeostasis, providing a suitable microenvironment for amine oxidation reactions [47].

Furthermore, the *urdA*-encoded uric acid reductase was identified in the LPWYP genome, serving as a critical branching regulatory node for this utilization pathway. Previous studies have shown that *urdA* catalyzes the reduction in uridine monophosphate to dihydrouridine monophosphate, thereby participating in the further metabolism or energy utilization of nitrogen-containing heterocyclic compounds [48]. This reaction not only expands the metabolic pathways of imidazole intermediates but may also function as an electron acceptor in energy metabolism under anaerobic conditions.

### 3.6. Strain Safety Analysis

#### 3.6.1. Nitroreductase, Indole, and Hemolysis Assays Combined with Genomic Analysis of Virulence Factors

**Figure 6:**
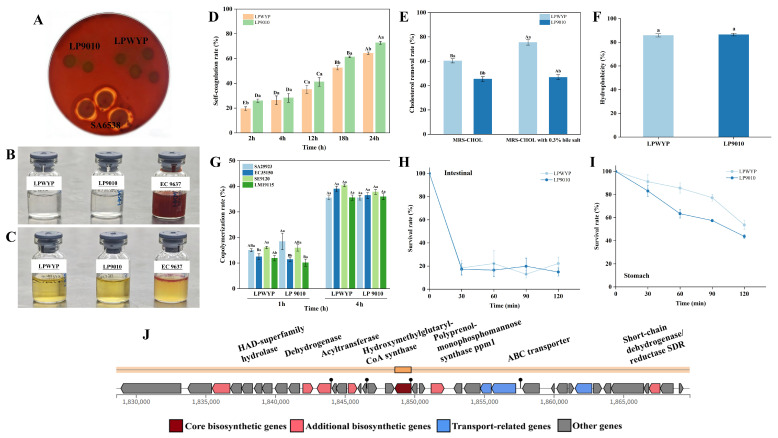
Analysis of the safety, tolerability, and adhesion properties of LPWYP. (**A**) shows the results of the hemolysis assay; (**B**) shows the results of the nitrite reductase assay; (**C**) shows the results of the indole assay; (**D**) shows the assessment of self-aggregation; (**E**) shows the assessment of cholesterol degradation; (**F**) shows cell surface hydrophobicity; (**G**) shows the measurement of aggregate adhesion; (**H**) artificial intestinal fluid assay; (**I**) artificial gastric fluid assay; (**J**) antimicrobial profile of LPWYP. Different lowercase letters indicate significant differences between the experimental and control groups (*p* < 0.05). Different uppercase letters indicate significant differences among groups (*p* < 0.05). LP9010 was used as the positive control. The labels on the small bottles in (**B**,**C**) are the exclusive marks of Guangdong Huan Kai Microbiological Technology Co., Ltd.

The safety of LPWYP was initially assessed through key phenotypic assays. In tests for harmful metabolite production, both LPWYP and the reference strain LP9010 yielded negative results for nitroreductase and indole (tryptophanase) activity, in contrast to the positive controls (Figure 6B,C). This indicates that LPWYP does not produce these specific harmful enzymes under the tested conditions [29].

Hemolytic activity was evaluated on blood agar. LPWYP formed colonies without any clearing zone, displaying a γ-hemolytic (non-hemolytic) phenotype identical to LP9010. This was in stark contrast to the clear β-hemolysis (complete hemolysis) exhibited by the positive control SA6538 (Figure 6A). These results confirm the non-hemolytic nature of strain LPWYP.

To complement the phenotypic safety assessment, the LPWYP genome was queried against the Virulence Factor Database (VFDB). A total of 110 genes (3.43% of the genome) showed homology to known virulence factors. Functional categorization revealed that most annotated genes were involved in processes common to probiotic bacteria, including immune modulation (41 genes), nutrition/metabolism (23 genes), and adhesion (11 genes)—functions that may contribute to intestinal colonization and microecological regulation rather than pathogenicity. As listed in Table 2, among the toxin-related genes identified, five putative exotoxin homologs were detected. Further analysis indicated that these genes primarily participate in transcriptional regulation of toxin synthesis (e.g., *cylR2*), bacterial self-protection mechanisms (e.g., *argK*), or encode indirect effector proteins with unique modes of action (e.g., *hlyA*/*TlyA*) [49,50]. One gene annotated as a hemolysin III (*hlyIII*) homolog warranted particular attention, as this family member encodes a pore-forming toxin in pathogenic Bacillus cereus. However, the phenotypic hemolysis assay conclusively demonstrated that LPWYP exhibits no visible hemolytic activity (γ-hemolysis), contrasting sharply with the complete hemolysis mediated by *hlyIII* in pathogenic strains. This discrepancy provides critical functional evidence that, despite the presence of an *hlyIII* homolog in the genome, the gene likely lacks functionality due to sequence divergence (<60% identity with known toxins), transcriptional silencing, or translational inactivation.

#### 3.6.2. Antibiotic Resistance Analysis

Antimicrobial susceptibility, a critical safety criterion for probiotic evaluation, was systematically assessed in strain LPWYP and the reference strain LP9010 using the Kirby–Bauer disk diffusion method, complemented by genomic annotation of antibiotic resistance genes against the Antibiotic Resistance Genes Database (ARDB).

As summarized in Table 3, LPWYP exhibited high susceptibility to β-lactams (amoxicillin, ampicillin), chloramphenicol, erythromycin, and tetracycline, indicating the absence of intrinsic resistance barriers to these clinically important antibiotics. The reference strain LP9010 was highly susceptible to ampicillin and intermediately susceptible to amoxicillin, chloramphenicol, and erythromycin. Notably, both strains displayed resistance to aminoglycosides (gentamicin, streptomycin, kanamycin), fluoroquinolones (norfloxacin), and vancomycin, as evidenced by zero inhibition zone diameters. In the species *Lactiplantibacillus plantarum*, vancomycin resistance is a typical intrinsic resistance; the mechanism involves the replacement of D-alanine at the terminal end of the cell wall peptidoglycan precursor with D-lactic acid, thereby preventing vancomycin from binding to its target [51]. This intrinsic resistance, based on species-specific metabolic characteristics, does not pose a risk of genetic transfer and is considered acceptable in safety assessments [52].

Genomic screening against the ARDB identified nine putative antibiotic resistance gene homologs in the LPWYP genome. Among these, several genes encoded efflux pump proteins potentially involved in resistance to deoxycholate, fosfomycin, lincosamides, and quinolones. A bacitracin resistance-associated undecaprenyl pyrophosphate phosphatase gene was also identified, exhibiting relatively high sequence identity (79.6%). Although genome screening identified some drug resistance gene fragments, such as *vanX* and *tet* families, they were mostly distributed in fragmented form and not linked to transposons or integrators, significantly reducing the risk of horizontal gene transfer [53]. This genomic configuration suggests that these fragments are unlikely to constitute functional, expressible, or transferable vancomycin resistance determinants.

In conclusion, LPWYP is susceptible to multiple clinically relevant antibiotics, including β-lactams, macrolides, tetracyclines, and chloramphenicol, with a susceptibility profile generally superior to that of reference strain LP9010. Although resistance to vancomycin and certain other antibiotics was observed phenotypically, genomic analysis revealed that the corresponding gene homologs lack integrity and are not associated with mobile elements, rendering horizontal transfer risk low. The remaining annotated resistance genes primarily target non-core or topical antibiotics (e.g., bacitracin, fosfomycin). Collectively, these findings support the safety of LPWYP with respect to antibiotic resistance, reinforcing its potential as a probiotic candidate.

### 3.7. Analysis of the Probiotic Properties of the Strain

#### 3.7.1. Cholesterol Degradation Capacity

In vitro assays demonstrated that LPWYP effectively degraded cholesterol in culture medium, with degradation activity significantly enhanced in the presence of 0.3% bile salts—a performance superior to that of reference strain LP9010 (Figure 6E). Genome annotation revealed the presence of bile salt hydrolase (BSH)-encoding genes (Table S3), which catalyze the deconjugation of bile salts. This enzymatic activity not only enhances strain tolerance to intestinal bile salts but also facilitates cholesterol removal through the formation of insoluble complexes with deconjugated bile acids, thereby promoting fecal excretion [54]. This phenotype–genotype correlation elucidates the potential mechanism by which LPWYP contributes to lipid metabolism regulation.

#### 3.7.2. Antimicrobial Activity

Agar diffusion assays revealed that cell-free culture supernatants of LPWYP exhibited pronounced inhibitory activity against *Escherichia coli*, *Staphylococcus aureus*, *Listeria monocytogenes*, and *Salmonella Typhimurium* (Table 4). In contrast, whole-cell suspensions produced only weak inhibition against *E. coli* and no detectable inhibition against the other three pathogens. This differential activity indicates that the antimicrobial function of LPWYP is primarily attributed to secreted metabolites, including organic acids or bacteriocin-like antimicrobial peptides, rather than to direct cell contact [55]. Genomic analysis identified multiple genes potentially involved in bacteriocin synthesis and stress-related metabolite production, supporting the observed secretory antimicrobial phenotype. Both the inhibition spectrum and intensity of LPWYP closely matched those of reference strain LP9010, demonstrating that LPWYP possesses secretory antimicrobial mechanisms comparable to this well-characterized strain.

Analysis of the LPWYP genome using AntiSMASH 7.0 revealed the presence of a gene cluster potentially associated with the synthesis of antimicrobial compounds; this cluster belongs to the type III polyketide synthases (PKS) (Figure 6J). T3PKS have relatively simple structures but diverse functions, often catalyzing the synthesis of phenolic derivatives or long-chain fatty acids with biological activities such as antimicrobial and anti-inflammatory effects [56]. Polyprenol-monophosphomannose synthase was also identified within this gene cluster, suggesting that LPWYP may be involved in the biosynthesis of complex glycolipids or cell wall-associated components.

#### 3.7.3. Adhesion and Intestinal Colonization Potential

The ability to adhere and colonize the intestinal tract is essential for probiotics to exert long-term beneficial effects. A series of in vitro assays revealed that LPWYP exhibited high auto-aggregation capacity and cell surface hydrophobicity (Figure 6D,F), traits that facilitate initial physical adherence to the intestinal mucosal surface. Furthermore, LPWYP demonstrated high co-aggregation rates with pathogenic bacteria including *E. coli* and *S. aureus*, suggesting potential for competitive pathogen exclusion through physical “aggregation-clearance” mechanisms [57] (Figure 6G). Genome analysis identified an abundance of environmental adaptation genes supporting these adhesive phenotypes, including those encoding heat shock proteins (*DnaK-DnaJ-GrpE*, *GroEL-GroES*) and cold shock protein *CspA* for temperature adaptation; various ABC transporters potentially involved in osmotic stress response; and universal stress proteins (USPs) along with ATP-dependent protease systems (*Clp*, *Lon*) for maintaining protein homeostasis [58]. This complex genetic network collectively constitutes the molecular basis for stable colonization and sustained survival of LPWYP in the dynamic intestinal environment.

#### 3.7.4. Gastrointestinal Environment Tolerance

Orally administered probiotics must withstand the harsh conditions of the gastrointestinal tract to reach the intestine in a viable state. In simulated gastrointestinal transit assays, LPWYP exhibited robust tolerance (Figure 6H,I). After 120 min exposure to simulated gastric fluid at pH 2.5, approximately 58% of cells remained viable. Genome analysis attributed this acid resistance to multiple encoded acid tolerance systems, including the F_0_F_1_-ATPase (atp operon) that pumps protons at the expense of ATP, and Na^+^/H^+^ antiporters (*nhaC*, *nhaK*) that assist in ion homeostasis [59,60]. Although viability decreased upon subsequent exposure to simulated intestinal fluid containing bile salts and digestive enzymes, tolerance was maintained through the synergistic action of several genomic determinants: bile salt detoxification mediated by BSH genes, osmotic adaptation via various ABC transporters, and repair of enzymatic damage by protease/chaperone systems. These integrated stress resistance mechanisms, encoded within the LPWYP genome, ensure that a fraction of viable cells successfully traverse the upper gastrointestinal tract, thereby enabling intestinal colonization and subsequent probiotic function.

## 4. Discussion and Conclusions

### 4.1. Potential Applications in the Food System

This study systematically characterized LPWYP, isolated from pineapple peel residues, by integrating phenotypic experiments with whole-genome sequencing. Strain LPWYP exhibited significant histamine-degrading capacity, as well as good gastrointestinal tolerance, antimicrobial activity, and preliminary probiotic properties. Safety assessments indicated that its antibiotic resistance profile aligns with the inherent characteristics of Lactobacillus, and no transferable acquired resistance genes were detected. Based on these characteristics, LPWYP holds clear application prospects in fermented food systems. It may be considered as a functional starter culture or adjunct culture for the production of fermented vegetables, dairy products, or fermented meat products, with the aim of simultaneously inhibiting pathogenic bacteria and degrading accumulated biogenic amines during fermentation. Future application studies should focus on inoculation strategies, interactions with indigenous microbial communities, and their impact on product sensory quality to facilitate the transition from laboratory potential to industrial application.

### 4.2. Limitations and Future Prospects

Although this study characterized the properties of LPWYP using multidimensional approaches, the following limitations remain. First, the elucidation of the histamine degradation mechanism relies primarily on genomic analysis. Although the presence of genes such as *cueO* and related aldehyde dehydrogenases supports the proposed PPU pathway, further studies (such as transcriptomics or proteomics) are needed to verify the expression levels of these genes under histamine stimulation. Second, the assessment of LPWYP’s probiotic potential is based on in vitro experiments; its colonization efficacy, immunomodulatory effects, and long-term safety in the human gastrointestinal tract still require verification through rigorous animal studies and human clinical trials.

### 4.3. Conclusions

In summary, we conducted a comprehensive analysis of the genomic and phenotypic characteristics of LPWYP to evaluate its safety and probiotic properties. The results indicate that LPWYP does not produce harmful metabolites, possesses good adhesion capacity and gastrointestinal tolerance, and carries a bacteriocin gene cluster. Most importantly, LPWYP exhibits favorable probiotic properties, making it suitable for food applications.

## Figures and Tables

**Table 1:** General genomic features of LPWYP.

Feature	LPWYP
Genome Size (bp)	3,336,829
GC content (%)	45.57
CDSs	3200
Plasmid	2
rRNA	16
tRNA	75
sRNA	4
TRF	86
CRISPR	2

**Table 2:** Prediction of virulence factor genes of LPWYP by VFDB (partial display).

Gene ID	Identity (%)	Description	VF_Type
GL000198	40.3	(cylR2) cytolysin regulator R2 [Cytolysin (VF0356)—Exotoxin (VFC0235)]	Exotoxin
GL000441	44.8	(*argK*) ornithine carbamoyltransferase [Phytotoxin phaseolotoxin (VF0917)—Exotoxin (VFC0235)]	Exotoxin
GL001179	50.3	(cysC1) adenylyl-sulfate kinase [Phytotoxin phaseolotoxin (VF0917)—Exotoxin (VFC0235)]	Exotoxin
GL001376	43.1	(hlyA) TlyA family RNA methyltransferase [Hemolysin (VF0879)—Exotoxin (VFC0235)]	Exotoxin
GL002806	42.9	(hlyIII) hemolysin III family protein [Hemolysin III (VF0655)—Exotoxin (VFC0235)]	Exotoxin

**Table 3:** Antibiotic sensitivity of LPWYP.

Antibiotics	Drug Content (μg/piece)	Inhibitory Diameter (mm)	
		LPWYP	LP9010
Amoxicillin	20	39.01 ± 0.75 ^Aa^ (H)	10.89 ± 0.34 ^Cb^ (I)
Chloramphenicol	30	34.61 ± 1.05 ^Ca^ (H)	13.23 ± 0.56 ^Bb^ (I)
Vancomycin	30	0 ^Bb^ (R)	0 ^Bb^ (R)
Erythromycin	15	28.28 ± 0.25 ^Da^ (H)	10.89 ± 0.23 ^Cb^ (I)
Gentamicin	10	0 ^Bb^ (R)	0 ^Bb^ (R)
Streptomycin	10	0 ^Bb^ (R)	0 ^Bb^ (R)
Ampicillin	10	37.65 ± 0.93 ^Ba^ (H)	22.89 ± 0.76 ^Aa^ (H)
Kanamycin	30	0 ^Bb^ (R)	0 ^Bb^ (R)
Tetracycline	30	21.92 ± 0.98 ^Ea^ (I)	8.76 ± 0.34 ^Db^ (I)
Norfloxacin	10	0 ^Bb^ (R)	0 ^Bb^ (R)

Antibiotic resistance diameter (mm): ≥15 mm indicates highly susceptible (H); 10–14 mm indicates intermediate susceptibility (I); <10 mm indicates low susceptibility (L); 0 mm indicates resistance (R). Distinct superscripted lowercase letters denotes significant difference between experimental group and control group (*p* < 0.05). Distinct superscripted uppercase letters denote significant difference between different groups (*p* < 0.05). LP9010 was used as the positive control.

**Table 4:** Antimicrobial effect of LPWYP.

Pathogenic Bacteria	Cell-Free Supernatant (mm)	Bacterial Suspension (mm)	Cell-Free Supernatant (mm)	Bacterial Suspension (mm)
	LPWYP		LP9010	
EC35150	10.31 ± 0.25 ^a^	5.43 ± 0.18 ^a^	10.25 ± 0.34 ^a^	5.31 ± 0.32 ^a^
SA25923	5.65 ± 0.56 ^a^	0 ^a^	5.52 ± 0.15 ^a^	0 ^a^
LM19115	4.45 ± 0.23 ^a^	0 ^a^	4.62 ± 0.18 ^a^	0 ^a^
ST14028	5.64 ± 0.67 ^a^	0 ^a^	5.43 ± 0.32 ^a^	0 ^a^

Different superscripted lowercase letters in the table peers represent significant differences between the two strains (supernatant and bacterial) (*p* < 0.05). LP9010 was used as the positive control.

## Data Availability

The raw whole-genome data and assembled sequence for LPWYP have been submitted to the GenBank database of the National Center for Biotechnology Information (NCBI), with BioProject accession number PRJNA1459682. The original contributions presented in this study are included in the article/Supplementary Materials. Further inquiries can be directed to the corresponding authors.

## Supplementary Materials

The following supporting information can be downloaded at: https://www.mdpi.com/article/10.3390/foods15101763/s1, Table S1. Histamine degradation rate. Table S2. Statistical table of genome coverage. Table S3. Analysis of tolerance gene of LPWYP. Figure S1. HPLC chromatograms of histamine degradation by the top 5 strains. A: Chromatogram of the standard histamine solution (500 mg L^−1^). B–F: Chromatograms of histamine after treatment with the top 5 degrading strains (No. 15, 37, 40, 46, and 43, respectively). Figure S2. Standard curve of histamine.
