# Supplementary material for: Screening and Characterization of Lactiplantibacillus plantarum WYP with Histamine-Degrading Activity: A Probiotic Candidate Assessed Based on Phenotyping Experiments and Whole-Genome Sequencing
Source: Foods. 2026 May 16;15(10):1763. doi: 10.3390/foods15101763 (PMC13205391; doi:10.3390/foods15101763)
Supplement: Supplementary file 1 [file foods-15-01763-s001.zip › foods-4291620-supplementary.pdf]

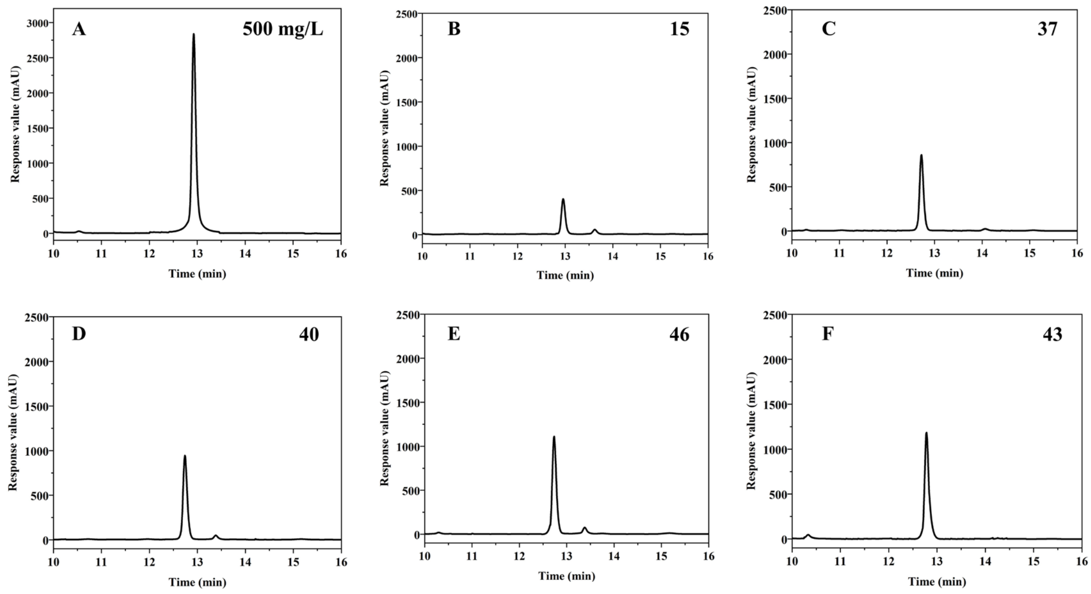

Figure S1. HPLC chromatograms of histamine degradation by the top 5 strains. A: Chromatogram of the standard histamine solution (500 mg L^{-1}). B-F: Chromatograms of histamine after treatment with the top 5 degrading strains (No. 15, 37, 40, 46, and 43, respectively).

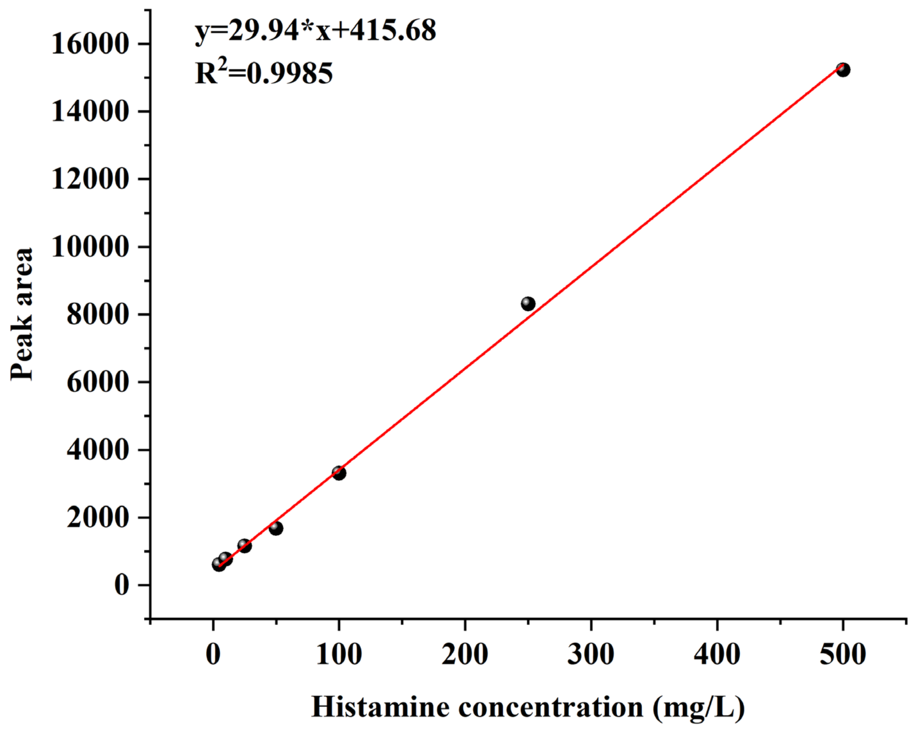

Figure S2. Standard curve of histamine.

Table S1. Histamine degradation rate

**Strains**	**Final histamine concentration (mg/L)**			**Histamine degradation rate (%)**
	**Parallel 1**	**Parallel 2**	**Parallel 3**	
15	74.87	20.59	225.15	78.63 ± 21.19[a]
46	193.49	216.44	202.16	59.01 ± 3.25[b]
37	90.74	262.72	213.1	64.65 ± 24.32[b]
40	222.77	179.04	196.33	59.82 ± 6.18[b]
18	199.95	294.24	314.07	46.12 ± 12.20[c]
26	329.98	191.10	408.56	38.02 ± 22.02[c]
21	345.25	345.02	171.11	42.57 ± 20.09[c]
43	304.61	286.17	182.63	48.44 ± 13.15[c]
33	278.77	211.13	316.92	46.21 ± 10.72[c]
30	267.72	343.03	312.63	38.44 ± 7.58[c]

Note: Different lowercase letters indicate significant differences among the samples ($p < 0.05$)

Table S2. Statistical table of genome coverage.

ChrID	Referance_size (bp)	Covered_length (bp)	Coverage (%)	Depth
Chromosome1	3250423	3250422	100	380
Plasmid1	44586	44585	100	430
Plasmid2	41820	41819	100	410
Total	3336829	3336826	100	381

Table S3. Analysis of tolerance gene of LPWYP

Gene ID	Gene name	Function
**Universal stress family protein**		
LPWYPGL000981	--	Nucleotide-binding universal stress protein
LPWYPGL001089	--	Nucleotide-binding universal stress protein
LPWYPGL001466	--	Nucleotide-binding universal stress protein
LPWYPGL001504	--	Nucleotide-binding universal stress protein
LPWYPGL001914	GSP13	general stress protein 13
LPWYPGL002015	--	Nucleotide-binding universal stress protein
LPWYPGL002278	--	Nucleotide-binding universal stress protein
LPWYPGL002358	--	Nucleotide-binding universal stress protein
LPWYPGL002475	--	Nucleotide-binding universal stress protein
LPWYPGL002564	--	Nucleotide-binding universal stress protein
LPWYPGL003095	--	Nucleotide-binding universal stress protein
**Proteases and chaperones**		
LPWYPGL000604	clpP	ATP-dependent protease ClpP, protease subunit
LPWYPGL000805	clpC	ATP-dependent Clp protease, ATP-binding subunit ClpA
LPWYPGL001050	clpE	ATP-dependent Clp protease, ATP-binding subunit ClpA
LPWYPGL001150	clpP	ATP-dependent protease ClpP, protease subunit
LPWYPGL001814	clpX	ATP-dependent protease Clp, ATPase subunit ClpX
LPWYPGL003025	clpX	ATP-dependent Clp protease, ATP-binding subunit ClpA
LPWYPGL001592	hslU	ATP-dependent protease HslVU (ClpYQ), ATPase subunit HslU
LPWYPGL001593	hslV, clpQ	ATP-dependent protease HslVU (ClpYQ), peptidase subunit
LPWYPGL001639	clpB	ATP-dependent Clp protease, ATP-binding subunit ClpA
LPWYPGL001828	K07177	Lon-like protease
**Heat-shock stress**		
LPWYPGL000116	HSP20	Small heat shock protein IbpA, HSP20 family； Hsp20/alpha crystallin family protein
LPWYPGL000449	hslR	ribosome-associated heat shock protein Hsp15
LPWYPGL002292	HSP20	Small heat shock protein IbpA, HSP20 family
LPWYPGL002856	HSP20	Small heat shock protein IbpA, HSP20 family
LPWYPGL000553	groES, HSPE1	co-chaperone GroES
LPWYPGL000554	groEL, HSPD1	Chaperonin GroEL (HSP60 family)
LPWYPGL000455	hslO	Redox-regulated molecular chaperone, HSP33 family
LPWYPGL001731	dnaJ	DnaJ-class molecular chaperone with C-terminal Zn finger domain；olecular chaperone DnaJ
LPWYPGL001732	dnaK, HSPA9	Molecular chaperone DnaK (HSP70)
LPWYPGL001733	GRPE	Molecular chaperone GrpE (heat shock protein HSP-70)
LPWYPGL001734	hrcA	Transcriptional regulator of heat shock response
**Cold-shock stress**		
LPWYPGL000030	cspA	Cold-shock protein

LPWYPGL000789	cspA	Cold-shock protein
LPWYPGL000977	cspA	Cold-shock protein
**Bile salt resistance**		
LPWYPGL002991	Bsh	bile salt hydrolase , choloylglycine Hydrolase
**Acid stress**		
LPWYPGL002034	ATPF1E, atpC	FoF1-type ATP synthase, epsilon subunit
LPWYPGL002035	ATPF1B, atpD	FoF1-type ATP synthase, beta subunit
LPWYPGL002036	ATPF1G, atpG	FoF1-type ATP synthase, gamma subunit
LPWYPGL002037	ATPF1A, atpA	FoF1-type ATP synthase, alpha subunit
LPWYPGL002038	ATPF1D, atpH	FoF1-type ATP synthase, delta subunit
LPWYPGL002039	ATPF0B, atpF	FoF1-type ATP synthase, membrane subunit b or b'
LPWYPGL002040	ATPF0C, atpE	FoF1-type ATP synthase, membrane subunit c/Archaeal/vacuolar-type H+-ATPase, subunit K
LPWYPGL002041	ATPF0A, atpB	FoF1-type ATP synthase, membrane subunit a
LPWYPGL000169	nhaC	Na+/H+ antiporter NhaC/MleN
LPWYPGL002844	nhaC	Na+/H+ antiporter NhaC/MleN
LPWYPGL000466	--	Na+/H+ antiporter NhaD or related arsenite permease
LPWYPGL000631	nhaK	NhaP-type Na+/H+ or K+/H+ antiporter
LPWYPGL002245	nhaK	NhaP-type Na+/H+ or K+/H+ antiporter
LPWYPGL002297	nhaK	NhaP-type Na+/H+ or K+/H+ antiporter
LPWYPGL002676	nhaK	NhaP-type Na+/H+ or K+/H+ antiporter
**Osmotic stress**		
LPWYPGL000018	oppA, mppA	ABC-type oligopeptide transport system, periplasmic component
LPWYPGL000080	oppA, mppA	ABC-type oligopeptide transport system, periplasmic component
LPWYPGL000087	cbiM	ABC-type Co2+ transport system, permease component
LPWYPGL000131	ecfA	ABC-type glutathione transport system ATPase component, contains duplicated ATPase domain
LPWYPGL000140	narA	ABC-type multidrug transport system, ATPase component
LPWYPGL000155	ganP, mdxF	ABC-type sugar transport system, permease component
LPWYPGL000156	ganQ, mdxG	ABC-type maltose transport system, permease component MalG
LPWYPGL000159	msmX/K, malK, sugC, ggtA, msiK	ABC-type sugar transport system, ATPase component MalK
LPWYPGL000175	oppA, mppA	ABC-type oligopeptide transport system, periplasmic component
LPWYPGL000176	oppA, mppA	ABC-type oligopeptide transport system, periplasmic component
LPWYPGL000193	ykoE	ABC-type thiamine/hydroxymethylpyrimidine transport system, permease component

LPWYPGL000261	ABC.CD.P	ABC-type transport system involved in lipoprotein release, permease component LolC
LPWYPGL000262	ABC.CD.A	ABC-type lipoprotein export system, ATPase component
LPWYPGL000281	potC	ABC-type spermidine/putrescine transport system, permease component II
LPWYPGL000282	potB	ABC-type spermidine/putrescine transport system, permease component I
LPWYPGL000283	potA	ABC-type Fe3+/spermidine/putrescine transport systems, ATPase component
LPWYPGL000290	ytrB	ABC-type multidrug transport system, ATPase component
LPWYPGL000303	metQ	ABC-type metal ion transport system, periplasmic component/surface antigen
LPWYPGL000304	metN	ABC-type methionine transport system, ATPase component
LPWYPGL000305	metI	ABC-type methionine transport system, permease component
LPWYPGL000310	tagG	ABC-type polysaccharide/teichoic acid/polyol phosphate export permease
LPWYPGL000311	tagH	ABC-type polysaccharide/polyol phosphate transport system, ATPase component
LPWYPGL000332	opuBD	ABC-type proline/glycine betaine transport system, permease component
LPWYPGL000333	opuA	ABC-type proline/glycine betaine transport system, ATPase component
LPWYPGL000364	blpA, lagD	ABC-type bacteriocin/lantibiotic exporters, contain an N-terminal double-glycine peptidase domain
LPWYPGL000399	ABC.ZM.A	ABC-type Mn2+/Zn2+ transport system, ATPase component
LPWYPGL000400	ABC.ZM.P	ABC-type Mn2+/Zn2+ transport system, permease component
LPWYPGL000541	phnE	ABC-type phosphate/phosphonate transport system, permease component
LPWYPGL000542	phnE	ABC-type phosphate/phosphonate transport system, permease component
LPWYPGL000543	phnC	ABC-type phosphate/phosphonate transport system, ATPase component
LPWYPGL000544	phnD	ABC-type phosphate/phosphonate transport system, periplasmic component
LPWYPGL000558	pstS	ABC-type phosphate transport system, periplasmic component
LPWYPGL000568	pstS	ABC-type phosphate transport system, periplasmic component
LPWYPGL000569	pstC	ABC-type phosphate transport system, permease component
LPWYPGL000570	pstA	ABC-type phosphate transport system, permease component
LPWYPGL000571	pstB	ABC-type phosphate transport system, ATPase component
LPWYPGL000572	pstB	ABC-type phosphate transport system, ATPase component
LPWYPGL000602	oppA, mppA	ABC-type oligopeptide transport system, periplasmic component
LPWYPGL000619	glnP	ABC-type amino acid transport/signal transduction system, periplasmic component/domain
LPWYPGL000620	glnQ	ABC-type polar amino acid transport system, ATPase component
LPWYPGL000685	peb1C, glnQ	ABC-type polar amino acid transport system, ATPase component
LPWYPGL000686	peb1A, glnH	ABC-type amino acid transport/signal transduction system, periplasmic component/domain
LPWYPGL000687	peb1B, glnP, glnM	ABC-type amino acid transport system, permease component
LPWYPGL000688	peb1B, glnP, glnM	ABC-type amino acid transport system, permease component

LPWYPGL000921	mntA	ABC-type Mn2+/Zn2+ transport system, ATPase component
LPWYPGL000922	mntB	ABC-type Mn2+/Zn2+ transport system, permease component
LPWYPGL000923	mntC	ABC-type Zn uptake system ZnuABC, Zn-binding component ZnuA
LPWYPGL000951	cydD	ABC-type transport system involved in cytochrome bd biosynthesis, ATPase and permease components
LPWYPGL000952	cydC	ABC-type transport system involved in cytochrome bd biosynthesis, fused ATPase and permease components
LPWYPGL001043	oppA, mppA	ABC-type oligopeptide transport system, periplasmic component
LPWYPGL001044	oppB	ABC-type dipeptide/oligopeptide/nickel transport system, permease component
LPWYPGL001045	oppC	ABC-type dipeptide/oligopeptide/nickel transport system, permease component
LPWYPGL001046	oppD	ABC-type dipeptide/oligopeptide/nickel transport system, ATPase component
LPWYPGL001047	oppF	ABC-type glutathione transport system ATPase component, contains duplicated ATPase domain
LPWYPGL001090	ugpC	ABC-type sugar transport system, ATPase component MalK
LPWYPGL001091	ugpA	ABC-type sugar transport system, permease component
LPWYPGL001092	ugpE	ABC-type glycerol-3-phosphate transport system, permease component
LPWYPGL001093	ugpB	ABC-type glycerol-3-phosphate transport system, periplasmic component
LPWYPGL001100	ABC-2.A	ABC-type multidrug transport system, ATPase component
LPWYPGL001190	ABC.CD.P	ABC-type lipoprotein export system, ATPase component
LPWYPGL001244	ecsA	ABC-type multidrug transport system, ATPase component
LPWYPGL001245	ecsB	Predicted ABC-type exoprotein transport system, permease component
LPWYPGL001260	ABC.FEV.S	ABC-type Fe3+-hydroxamate transport system, periplasmic component
LPWYPGL001261	ABC.FEV.A	ABC-type cobalamin/Fe3+-siderophores transport system, ATPase component
LPWYPGL001262	ABC.FEV.P	ABC-type Fe3+-siderophore transport system, permease component
LPWYPGL001379	opuA	ABC-type proline/glycine betaine transport system, ATPase component
LPWYPGL001380	opuBD	ABC-type proline/glycine betaine transport system, permease component
LPWYPGL001381	opuC	Periplasmic glycine betaine/choline-binding (lipo)protein of an ABC-type transport system (osmoprotectant binding protein)
LPWYPGL001382	opuBD	ABC-type proline/glycine betaine transport system, permease component
LPWYPGL001415	ABC-2.A	ABC-type multidrug transport system, ATPase component
LPWYPGL001501	metN	ABC-type methionine transport system, ATPase component
LPWYPGL001502	metI	ABC-type methionine transport system, permease component
LPWYPGL001503	metQ	ABC-type metal ion transport system, periplasmic component/surface antigen
LPWYPGL001507	afuA, fbpA	ABC-type Fe3+ transport system, periplasmic component
LPWYPGL001508	afuC, fbpC	ABC-type Fe3+/spermidine/putrescine transport systems, ATPase component
LPWYPGL001549	ABC-2.A	ABC-type multidrug transport system, ATPase component
LPWYPGL001644	drrB	ABC-type multidrug transport system, permease component
LPWYPGL001645	drrA	ABC-type multidrug transport system, ATPase component

LPWYPGL001678	ABC-2.P	ABC-type multidrug transport system, permease component
LPWYPGL001679	ABC-2.A	ABC-type multidrug transport system, ATPase component
LPWYPGL001684	ABC-2.A	ABC-type multidrug transport system, ATPase component
LPWYPGL001685	ABC-2.P	ABC-type transport system involved in multi-copper enzyme maturation, permease component
LPWYPGL001688	ytrB	ABC-type uncharacterized transport system, ATPase component
LPWYPGL001777	ABC.SN.A	ABC-type nitrate/sulfonate/bicarbonate transport system, ATPase component
LPWYPGL001778	ABC.SN.P	ABC-type anion transport system, duplicated permease component
LPWYPGL001808	artR, artM	ABC-type polar amino acid transport system, ATPase component
LPWYPGL001809	artP, artI	ABC-type amino acid transport/signal transduction system, periplasmic component/domain
LPWYPGL001983	ABC.PA.S	ABC-type amino acid transport/signal transduction system, periplasmic component/domain
LPWYPGL001984	ABC.PA.A	ABC-type polar amino acid transport system, ATPase component
LPWYPGL001985	ABC.PA.P	ABC-type amino acid transport system, permease component
LPWYPGL002024	metQ	ABC-type metal ion transport system, periplasmic component/surface antigen
LPWYPGL002025	metI	ABC-type methionine transport system, permease component
LPWYPGL002026	metN	ABC-type methionine transport system, ATPase component
LPWYPGL002059	efrB, efrF	ABC-type multidrug transport system, ATPase and permease component
LPWYPGL002060	efrA, efrE	ABC-type multidrug transport system, ATPase and permease component
LPWYPGL002155	abcA, bmrA	ABC-type multidrug transport system, ATPase and permease component
LPWYPGL002157	ABC.CD.P	ABC-type lipoprotein export system, ATPase component
LPWYPGL002191	K01989	ABC-type uncharacterized transport system, periplasmic component
LPWYPGL002192	K05832	ABC-type uncharacterized transport system, permease component
LPWYPGL002193	K05833	ABC-type uncharacterized transport system, ATPase component
LPWYPGL002250	ABC-2.A	ABC-type uncharacterized transport system, ATPase component
LPWYPGL002352	ABC.CD.A	ABC-type lipoprotein export system, ATPase component
LPWYPGL002353	ABC.CD.P	Predicted ABC-type transport system involved in lysophospholipase L1 biosynthesis, permease component
LPWYPGL002356	ABC-2.A	ABC-type uncharacterized transport system, ATPase component
LPWYPGL002382	ABC-2.P	ABC-type multidrug transport system, permease component
LPWYPGL002383	ABC-2.A	ABC-type multidrug transport system, ATPase component
LPWYPGL002425	ABC.CD.P	ABC-type transport system involved in lipoprotein release, permease component LolC
LPWYPGL002426	ABC.CD.A	ABC-type lipoprotein export system, ATPase component
LPWYPGL002451	ABC-2.A	ABC-type uncharacterized transport system, ATPase component
LPWYPGL002452	ABC-2.P	ABC-type Na+ efflux pump, permease component NatB
LPWYPGL002458	ABC.CD.P	ABC-type antimicrobial peptide transport system, permease component
LPWYPGL002459	ABC.CD.A	ABC-type lipoprotein export system, ATPase component
LPWYPGL002484	mdlB, smdB	ABC-type multidrug transport system, ATPase and permease component
LPWYPGL002484	mdlA, smdA	ABC-type multidrug transport system, ATPase and permease component
LPWYPGL002539	abcA, bmrA	ABC-type multidrug transport system, ATPase and permease component

LPWYPGL002543	K05833	ABC-type uncharacterized transport system, ATPase component
LPWYPGL002544	K05832	ABC-type uncharacterized transport system, permease component
LPWYPGL002545	K01989	ABC-type uncharacterized transport system, periplasmic component
LPWYPGL002553	livF	ABC-type branched-chain amino acid transport system, ATPase component LivF
LPWYPGL002554	livG	ABC-type branched-chain amino acid transport system, ATPase component LivG
LPWYPGL002555	livM	ABC-type branched-chain amino acid transport system, permease component
LPWYPGL002556	livH	Branched-chain amino acid ABC-type transport system, permease component
LPWYPGL002557	livK	ABC-type branched-chain amino acid transport system, periplasmic component
LPWYPGL002570	ABC.CD.P	ABC-type lipoprotein export system, ATPase component
LPWYPGL002588	ABC.ZM.S	ABC-type Zn uptake system ZnuABC, Zn-binding component ZnuA
LPWYPGL002605	patB, rscB, lmrC, satB	ABC-type multidrug transport system, ATPase and permease component
LPWYPGL002606	patA, rscA, lmrC, satA	ABC-type multidrug transport system, ATPase and permease component
LPWYPGL002659	fhuD, ftsB, siuD	ABC-type Fe3+-hydroxamate transport system, periplasmic component
LPWYPGL002660	fhuA, ftsA, siuA	ABC-type cobalamin/Fe3+-siderophores transport system, ATPase component
LPWYPGL002661	fhuB, ftsC, siuB	ABC-type Fe3+-siderophore transport system, permease component
LPWYPGL002662	fhuG, ftsD, siuG	ABC-type Fe3+-siderophore transport system, permease component
LPWYPGL002695	ABC.CD.A	ABC-type lipoprotein export system, ATPase component
LPWYPGL002745	tcyA, tcyJ, fliY	ABC-type amino acid transport/signal transduction system, periplasmic component/domain
LPWYPGL002746	tcyB, tcyL	ABC-type amino acid transport system, permease component
LPWYPGL002747	tcyC, tcyN	ABC-type polar amino acid transport system, ATPase component
LPWYPGL002748	tcyA, tcyJ, fliY	ABC-type amino acid transport/signal transduction system, periplasmic component/domain
LPWYPGL002757	STAR2, fetB	ABC-type iron transport system FetAB, permease component
LPWYPGL002758	STAR1, fetA	ABC-type iron transporter FetAB, ATPase component
LPWYPGL002821	ABC.ZM.S	ABC-type Zn uptake system ZnuABC, Zn-binding component ZnuA
LPWYPGL003069	msmX/K, malK, sugC, ggtA, msiK	ABC-type sugar transport system, ATPase component MalK
LPWYPGL003075	lplA	ABC-type glycerol-3-phosphate transport system, periplasmic component
LPWYPGL003076	lplC	ABC-type glycerol-3-phosphate transport system, permease component
LPWYPGL003077	lplB	ABC-type polysaccharide transport system, permease component
LPWYPGL003080	mdlA, smdA	ABC-type multidrug transport system, ATPase and permease component

LPWYPGL003081	mdlB, smdB	ABC-type multidrug transport system, ATPase and permease component
LPWYPGL003113	oppA, mppA	ABC-type oligopeptide transport system, periplasmic component
LPWYPGL002836	TC.BCT	Choline-glycine betaine transporter
**Oxidative stress**		
LPWYPGL000582	nox2	NADH oxidase
LPWYPGL000588	nox2	NADH oxidase
LPWYPGL001675	nox2	NADH oxidase
LPWYPGL002922	nox2	NADH oxidase
LPWYPGL003019	katE, CAT, catB, srpA	Catalase
LPWYPGL000194	gpx, btuE, bsaA	glutathione peroxidase
LPWYPGL002907	yfeX	dyp-type peroxidase
LPWYPGL000206	TXN, trxA	thioredoxin
LPWYPGL001950	TXN, trxA	thioredoxin
LPWYPGL002263	TXN, trxA	thioredoxin
LPWYPGL002913	TXN, trxA	thioredoxin
LPWYPGL000051	arsC	arsenate reductase (thioredoxin)
LPWYPGL000583	trxB, TRR	thioredoxin reductase (NADPH)
LPWYPGL001994	tpx	thioredoxin-dependent peroxiredoxin
LPWYPGL000334	GSR, gor	glutathione reductase (NADPH)
LPWYPGL001036	GSR, gor	glutathione reductase (NADPH)
LPWYPGL001577	GSR, gor	glutathione reductase (NADPH)
LPWYPGL002789	GSR, gor	glutathione reductase (NADPH)
LPWYPGL000240	mntH	manganese transport protein
LPWYPGL000399	ABC.ZM.A	zinc/manganese transport system ATP-binding protein
LPWYPGL000400	ABC.ZM.P	zinc/manganese transport system permease protein
LPWYPGL000921	mntA	manganese transport system ATP-binding protein
LPWYPGL000922	mntB	manganese transport system permease protein
LPWYPGL000923	mntC	manganese transport system substrate-binding protein
LPWYPGL001069	mntH	manganese transport protein
LPWYPGL002563	mntH	manganese transport protein
LPWYPGL002588	ABC.ZM.S	zinc/manganese transport system substrate-binding protein
LPWYPGL002821	ABC.ZM.S	zinc/manganese transport system substrate-binding protein
LPWYPGL000078	K09861	DNA-binding protein YaaA associated with the oxidative stress response
